# Use of Cigarettes With Flavor-Changing Capsules Among Smokers in the United Kingdom: An Online Survey

**DOI:** 10.1093/ntr/nty173

**Published:** 2018-08-24

**Authors:** Crawford Moodie, Anne Marie MacKintosh, James F Thrasher, Ann McNeill, Sara Hitchman

**Affiliations:** 1 Centre for Tobacco Control Research, Institute for Social Marketing, School of Health Sciences and Sport, University of Stirling, Stirlingshire, UK; 2 UK Centre for Tobacco and Alcohol Studies, Nottingham, UK; 3 Department of Health Promotion, Education, and Behavior, Arnold School of Public Health, University of South Carolina, Columbia, SC; 4 National Addiction Centre, Institute of Psychiatry, Psychology and Neuroscience, King’s College London, London, UK

## Abstract

**Introduction:**

Cigarettes with flavor-changing capsules in the filter have experienced phenomenal global growth in the last decade. We explore sociodemographic and smoking-related factors associated with using capsule cigarettes, how frequently users burst the capsule, and reasons for using them.

**Methods:**

An online survey was conducted in the United Kingdom between April and May 2016 with 6234 factory-made and/or hand-rolled cigarette smokers. This analysis focuses on 3620 factory-made cigarette smokers, aged 18 years and over, who had smoked in the past month.

**Results:**

Thirteen percent smoked capsule cigarettes, with younger smokers more likely than older smokers to do so. Capsule use was significantly more common among White non-British than White British and among those planning to quit in the next 6 months than those not planning to quit. Most capsule users who crushed the capsule did so always (51%) or most of the time (18%), with more frequent crushing of capsules more common among females, younger and middle-aged participants, White British, and those with a lower score on the Heaviness of Smoking Index. The most common reasons for using capsule cigarettes were that they taste better (52%), are smoother (41%), provide a choice of flavors (32%), and the enjoyment of clicking the capsule (25%). Capsule and noncapsule smokers did not differ significantly in their perceptions of the harmfulness of their brand relative to other brands.

**Conclusions:**

Our study provides an insight into how and why smokers of capsule cigarettes use these products, with the key drivers of use being taste, flavor choice, and interactivity.

**Implications:**

Cigarettes with capsules in the filter that can be burst to change the flavor have experienced remarkable growth since being introduced in 2007, but little is known about how and why smokers use these products. Thirteen percent of factory-made cigarette smokers in our sample smoked a brand with a capsule in the filter, with approximately two-thirds crushing the capsule all or most of the time. Capsule use was more likely among younger participants. The main reasons for smoking capsule cigarettes were related to how they taste, having a choice of flavors, and enjoyment of clicking the capsule (interactivity).

## Introduction

With most countries having implemented comprehensive bans on tobacco advertising, promotion, and sponsorship, packaging has taken on a more prominent marketing role. Tobacco companies do not have full control over the appearance of the pack, however, with more than 100 countries requiring pictorial warnings on packs,^1^ and a growing number of countries planning to introduce plain (or standardized) packaging, with Australia, France, the United Kingdom, New Zealand, and Norway having already done so. However, even in these countries, tobacco companies continue to promote their products, with the head of business development for British American Tobacco (BAT) explaining that innovation, and the product itself, become more important as a result of legislation requiring large pictorial warnings or plain packaging.^2^ By far, the most successful example of product innovation in recent times is the inclusion of flavor-changing capsules within the filters of cigarettes, described by a BAT spokesperson as the “biggest innovation in cigarettes since the filter.”^3^

The use of rupturable capsules within cigarettes dates back to the 1960s, although at that time capsules contained water, rather than flavoring properties, as the water was intended to soften the filter material when the cigarette was lit.^4^ The first flavor-changing capsules were introduced in Japan in 2007 and are now available in most markets. BAT, for instance, reported selling capsules in 116 markets in 2015.^5^ As the global footprint of these products has grown, so too has the product range. Since the first menthol capsules were brought to market, tobacco companies have introduced a panoply of new flavors, for instance, green tea flavor in China and whisky flavor in Japan,^6^ double capsules (two different flavor capsules within one filter), and also mixed packs (four or five different flavors within one pack).^7^

The capsule segment grew more than fourfold between 2011 and 2015^8^ and now has approximately 1.2% of the global cigarette market, with further growth predicted.^8^ While the three leading markets for capsules, in terms of percentage of total cigarette market share, are in Latin America, and the leading market by volume sales is Japan,^8^ the capsule segment has steadily grown in the United Kingdom. The first capsule variant was brought to the UK market in December 2011,^9^ with the capsule segment now accounting for just over 10% of the cigarette category.^10^

Despite the global success of capsule cigarettes, only a handful of studies have explored use of these products. Three focus group studies have been conducted in Scotland^11,12^ and the United States.^13^ In Scotland, among a sample of 12–24-year-old female nonsmokers and occasional smokers, capsule cigarettes were viewed very positively and regarded as a “cool invention,” with their novelty and the option to change the flavor thought to enhance their appeal.^11^ In a study with 18–24-year-old menthol smokers in the United States, several participants reported having tried a capsule brand variant (Camel Crush), which was viewed as fun and appealing for newer smokers, with crushing the capsule considered enjoyable.^13^ A second focus group study in Scotland, with smokers aged 16 and over, found that awareness, use, and appeal of capsules were greatest among younger adults (16–35 years).^12^ Those who perceived capsules positively mentioned multiple benefits, such as better taste, the ability to burst the capsule, convenience of being able to share cigarettes among menthol and nonmenthol smokers, fresher breath, reduced smell, and greater discretion. It was suggested that capsule cigarettes would encourage nonsmokers to experiment with smoking and discourage smokers from quitting.^12^

Four surveys have also explored the use of capsule cigarettes. In the Australian School Students Alcohol and Drug Survey with 12–17-year olds, more than half of past month smokers reported trial of a capsule cigarette (51.8%), more likely females than males.^14^ A survey with 11–16-year-old secondary school students in Mexico found that packs of capsule cigarettes were rated as more attractive than packs of noncapsule cigarettes, with greater interest in trying the capsule cigarettes.^15^ An online survey with adult smokers in the United States, Mexico, and Australia found that use of these products increased between 2012 and 2014 in Mexico (6% to 14%) and Australia (0.1% to 3%) but not significantly so in the United States (about 4% at each wave).^16^ This study also explored how these products are being used, with half of smokers in Mexico (52%) and approximately a third of smokers in Australia (30%) and the United States (37%) indicating that they always crushed the capsule; a smaller percentage reported never crushing the capsule (4% in Mexico, 5% in the United States, 16% in Australia). Preference for flavor capsules was much higher among younger than older adults in Australia and the United States and those who smoked capsule cigarettes perceived greater stylishness, taste, and relatively lower harm for their brand compared with those who smoked noncapsule cigarettes.^16^A survey in the United States found that use of flavor capsules among 18–44-year-old smokers was the highest among 18–24-year olds and decreased with age.^17^ Capsule smokers were less likely to be daily smokers than noncapsule smokers, with taste, satisfaction, and price the most common reasons for using capsules. Unlike previous research in the United States,^16^ capsule smokers were not less likely than noncapsule smokers to perceive their usual product as less harmful than other products.^17^

In this study, we explore sociodemographic and smoking-related factors of capsule and noncapsule cigarette smokers in the United Kingdom and how and why capsule smokers use these products.

## Methods

### Design and Sample

An online survey called the “Adult Tobacco Policy Survey” was conducted between April 20 and May 16, 2016 with smokers aged 16 and over (*N* = 6234) in the United Kingdom. The survey was undertaken by YouGov using a sample recruited from their panel of 816 300 people aged 16 and over. YouGov panel members are recruited from various sources, including advertising and partnerships with websites. The sample was weighted by age, gender, government office region, and tobacco consumption to represent the national profile of smokers aged 16 and over in the United Kingdom, which was based on the Opinions and Lifestyle Survey and Integrated Household Survey. Table 1 shows the full sample before and after weighting. The unweighted sample matched the national profile of smokers in terms of ethnicity, Heaviness of Smoking Index (HSI), and quit intentions, but males and young adults (aged 16–35 years) were under-represented. Weighting brought the sample in line with national profiles in terms of gender and age.

**Table 1.** Sample Characteristics of Full Sample of Smokers

Base: all smokers (16 and over)	Unweighted (*N* = 6234)		Weighted(*N* = 6234)	
	*n*	%	*N*	%
Gender				
Male	2889	46	3247	52
Female	3345	54	2987	48
Age group				
16–24	623	10	1099	18
25–34	1188	19	1331	21
35–44	1259	20	1062	17
45–54	1321	21	1159	19
55+	1843	30	1583	25
Social grade				
ABC1	3584	57	3707	59
C2DE	2472	40	2346	38
Not stated	178	3	181	3
Ethnicity				
White British	5500	88	5452	87
Other White background	352	6	374	6
Other	318	5	342	5
White and Black Caribbean	26	<0.5	27	<0.5
White and Black African	15	<0.5	17	<0.5
White and Asian	44	1	52	1
Any other mixed background	43	1	43	1
Indian	47	1	52	1
Pakistani	26	<0.5	38	1
Bangladeshi	11	<0.5	11	<0.5
Any other Asian	6	<0.5	5	<0.5
Black Caribbean	25	<0.5	22	<0.5
Black African	18	<0.5	20	<0.5
Any other Black	4	<0.5	3	<0.5
Chinese	14	<0.5	13	<0.5
Other ethnic group	39	1	40	1
Not stated	64	1	66	1
Heaviness of Smoking Index (HSI, 0 = light, 6 = heavy)				
0	1928	31	2128	34
1	746	12	785	13
2	1098	18	1090	17
3	1420	23	1319	21
4	651	10	559	9
5	217	3	176	3
6	78	1	63	1
Not stated	96	2	112	2
Whether planning to quit in next 6 months				
No	4162	67	4154	67
Yes	2072	33	2080	33

## Measures

### Demographics

Participants reported their age, gender, social grade (occupation of chief income earner within household), and ethnicity. Ethnicity was coded against 15 categories, but with few participants endorsing many of these, it was recoded into three categories (White British, White non-British, other) for analysis (see Table 1).

### Smoking Behavior

Participants were asked “Which of the following best applies to you? Please note cigarettes refer to those that are factory-made (packet) and also those that are hand-rolled (rolling tobacco). Cigarettes do not include electronic cigarettes or vaping devices.” The response options were “I smoke cigarettes (including hand-rolled) every day,” “I smoke cigarettes (including hand-rolled), but not every day,” “I do not smoke cigarettes at all, but I do smoke tobacco of some kind (e.g. Pipe, cigar or shisha),” “I have stopped smoking completely in the last year,” “I stopped smoking completely more than a year ago,” “I have never been a smoker,” and “Don’t know.” Nondaily cigarette smokers were subsequently asked: “Can we just confirm, how often do you currently smoke cigarettes (either factory-made or hand-rolled)?”, with response options “At least once a week,” “Less than once a week, but at least once a month,” “Less than once a month, but at least once in the last three months,” “I have not smoked cigarettes in the last three months,” and “Don’t know.” The inclusion criteria for the study were that participants had smoked cigarettes in the last three months.

The HSI^18^ was used to assess time to first cigarette and daily consumption. To measure consumption, smokers were asked how many cigarettes (either factory-made or hand-rolled) they smoked in a typical day, week, or month, depending on their response to questions on frequency of smoking. To assess quit behavior, participants were asked “Are you planning to quit smoking…” with response options “Within the next month,” “Between 1 and 6 months from now,” “Sometime in the future, beyond 6 months,” “Not planning to quit,” and “Don’t know.”

### Brand Smoked

Participants were asked whether they smoked more factory-made cigarettes or rolling tobacco, and on the basis of their response to this question if they had a brand of factory-made cigarettes (or rolling tobacco) that they usually smoked, with response options “Yes,” “No,” and “Don’t know.” For those who did not have a usual brand, they were asked what brand they currently smoked.

### Capsule Use

Participants were asked if their cigarette brand had a capsule in the filter which can be burst to change the flavor, with response options “Yes,” “No,” and “Don’t know.” This question was only asked to those who smoked factory-made cigarettes, and not those who only smoked rolling tobacco, given that there were no capsule brands of rolling tobacco on the UK market.

### Frequency and Timing of Bursting Capsule and Reasons for Use

Participants were asked “How often do you crush the capsule within the filter?” (Never; Rarely; Sometimes; Most of the time; Always; Don’t know).^16^ To explore reasons for use, they were asked “Why do you use capsule cigarettes?” with eight response options: “They taste better than regular cigarettes,” “They are smoother on my airways (i.e. throat and lungs) than regular cigarettes,” “I enjoy clicking the capsule,” “They are novel,” “They are more interesting than regular cigarettes,” “My friends, family or work colleagues use or recommended them,” “I like having a choice of flavours,” “My brand only has capsule cigarettes.” There was also an “Other” and “Don’t know” option. Participants could check all reasons that applied.

### Harm

Both capsule and noncapsule smokers were asked “Is your usual (current) brand of cigarettes a little less harmful, no different, or a little more harmful, compared with other brands?” (Little less harmful than other brands; No different; Little more harmful than other brands; Don’t know).

### Procedure

YouGov employ an active sampling method, drawing a subsample from their panel that is intended to be representative of the target sample. Randomly selected panel members were invited by e-mail to participate in this survey, with a link provided to do so; only those invited to participate could do so, and they were not informed in the e-mail invite about the topic area so as to minimize opt-out on this basis. A total of 13930 invitations were sent to smokers whose profiling data matched the recruitment criteria. Of the 8758 who clicked on the survey link, 1599 were screened out as they indicated that they did not meet the inclusion criteria, ie, that they smoked cigarettes in the last 3 months. Of the 7159 who completed the survey, there were 665 noncompleters, and 260 were removed by YouGov for data quality issues (eg, straight-lining, or completing the survey in less than half the average median time), leaving 6234 participants. Participants received a modest incentive (£2) for taking part. An information page was provided at the start of the survey, and explicit consent was required before participation. The School of Health Sciences and Sport Ethics Committee at the University of Stirling granted ethical approval.

### Analysis

Data were analyzed using SPSS Version 21. Analysis focused on the 3620 smokers aged 18 and over whose usual brand was factory-made cigarettes and who had smoked in the past month; we only included past month smokers to be consistent with previous research^16^ and excluded 16–17-year olds given the small sample size (*n* = 23). For characteristics of the analytic sample, see Table 2. Descriptive data were weighted by age, gender, government office region, and tobacco consumption to reflect smokers in the United Kingdom. Logistic regression was used to estimate associations between preference for brands with capsules [smoke a capsule brand (1) vs. do not smoke a capsule brand (0)] and demographic and smoking-related variables. For smokers of capsule brands, logistic regression assessed associations between frequency of crushing the capsule [always or most of the time (1) vs. rarely or sometimes (0), excluding those who never crushed the capsule] and demographic and smoking-related variables. Variables included in each model were gender, age, social grade, ethnicity, HSI, and plans to quit in the next 6 months. Age and HSI were included as categorical variables using the “difference contrast” in the SPSS v21 logistic regression command. This enabled each category of the predictor variable, with the exception of the first, to be compared with the average effect of the previous categories. For example, 25–34-year olds were compared with 18–24-year olds, 35–44-year olds were compared with 18–34-year olds, etc. Because of small cell sizes, HSI codes 4–6 were combined into one category and compared with HSI codes 0–3. Including HSI as a categorical variable also enabled the “not stated responses” to be included in the analysis. Logistic regression was run on unweighted data as the models controlled for demographic and smoking-related variables. A Mann–Whitney U test was used to examine whether capsule users differed from noncapsule users in their perceptions of the harm from their brand of cigarettes. This was run on weighted data as no other variables were being controlled for.

**Table 2.** Sample Characteristics of Factory-Made Cigarette Smokers, Aged 18 and Over, Who Have Smoked in the Last Month

Base: factory-made cigarette smokers (18 and over) who have smoked in the last month	Unweighted (*N* = 3620)		Weighted (*N* = 3548)	
	*n*	%	*N*	%
Gender				
Male	1585	44	1750	49
Female	2035	56	1798	51
Age group				
18–24	286	8	513	14
25–34	650	18	722	20
35–44	703	19	592	17
45–54	789	22	695	20
55+	1192	33	1026	29
Social grade				
ABC1	2209	61	2221	63
C2DE	1315	36	1232	35
Not stated	96	3	94	3
Ethnicity				
White British	3204	89	3110	88
Other White background	200	6	207	6
Other	183	5	198	6
White and Black Caribbean	13	<0.5	13	<0.5
White and Black African	6	<0.5	7	<0.5
White and Asian	24	1	28	1
Any other mixed background	28	1	28	1
Indian	35	1	39	1
Pakistani	17	<0.5	24	1
Bangladeshi	5	<0.5	6	<0.5
Any other Asian	2	<0.5	1	<0.5
Black Caribbean	17	<0.5	15	<0.5
Black African	8	<0.5	8	<0.5
Any other Black	4	<0.5	3	<0.5
Chinese	6	<0.5	6	<0.5
Other ethnic group	18	<0.5	19	<0.5
Not stated	33	1	33	1
Heaviness of Smoking Index (HSI, 0 = light, 6 = heavy)				
0	1097	30	1189	34
1	447	12	461	13
2	665	18	642	18
3	850	23	780	22
4	366	10	308	9
5	109	3	85	2
6	39	1	33	1
Not stated	47	1	49	1
Whether planning to quit in next 6 months				
No	2267	63	2212	62
Yes	1353	37	1335	38

## Results

### Sample Characteristics

Table 2 shows the characteristics for the analytic sample. After weighting, the sample contained 49% males, with those aged 55 years and over accounting for the highest proportion of the age groups examined (29%). Approximately two-thirds (63%) were middle-class (social grade ABC1) and most identified as White British (88%). The overall mean HSI was 1.7 (SD = 1.5).

### Prevalence of Capsule Use Among Factory-Made Cigarette Smokers

Thirteen percent indicated that they smoked a capsule brand. When demographic and smoking-related variables were controlled for, prevalence of capsule use did not differ significantly by gender, social grade, or HSI (Table 2). However, each age group was less likely than the younger age group(s) to smoke capsule cigarettes, eg, Adj OR = 0.463, *p* < .001 for 25–34-year olds compared with 18–24-year olds; Adj OR = 0.357, *p* < .001 for those aged 55+ compared with 18–54-year olds, see Table 3. Capsule use was more likely among White non-British than White British (Adj OR = 1.849, *p* = .002) and among those planning to quit smoking in the next 6 months than those not planning to quit in the next 6 months (Adj OR = 1.742, *p* < .001).

**Table 3.** Logistic Regression of Association Between Demographic and Smoking-Related Variables and Whether or Not They Smoke a Brand With Capsule

Base: factory-made cigarette smokers (18 and over), who have smoked in the last month (3620, unweighted)	1 = Brand has capsule (405)			
	0 = Brand does not have capsule (3215)			
	Adj OR^a^	95% CI lower	95% CI upper	*p*
Gender				
Male	ref			
Female	1.151	0.923	1.437	.212
Age group				<.001
18–24	ref			
25–34 vs. 18–24	0.463	0.333	0.643	<.001
35–44 vs. 18–34	0.406	0.303	0.543	<.001
45–54 vs. 18–44	0.325	0.237	0.446	<.001
55+ vs. 18–54	0.357	0.267	0.476	<.001
Social grade				.604
ABC1	ref			
C2DE	1.109	0.877	1.402	.389
Not stated	0.864	0.430	1.737	.682
Ethnicity				.010
White British	ref			
Other white background	1.849	1.259	2.717	.002
Other	1.163	0.761	1.776	.490
Not stated	0.456	0.105	1.987	.298
Heaviness of Smoking Index (HSI, 0 = light, 6 = heavy)				.565
0	ref			
1 vs. 0	0.893	0.632	1.261	.521
2 vs. (0–1)	0.894	0.654	1.223	.485
3 vs. (0–2)	0.830	0.621	1.109	.207
4–6 vs. (0–3)	0.873	0.602	1.268	.477
Not stated vs. (0–6)	1.136	0.479	2.695	.772
Whether planning to quit in next 6 mo				
No	ref			
Yes	1.742	1.400	2.166	<.001
	Test of model coefficients: *χ*^2^ = 223.566, *df* = 16, *p* < .001. Nagelkerke *R*^2^ = 0.119.			
	Hosmer & Lemeshow *χ*^2^ = 8.935, *df* = 8, *p* = .348.			

95% CI = 95% confidence interval; Adj OR = adjusted odds ratio; ref = reference category.

^a^Adjusted for all other variables in the model.

### Frequency of Crushing the Capsule

Among capsule smokers, about half (51%) always crushed the capsule, 18% did so most of the time, 12% sometimes, and 9% rarely. Almost one in 10 (9%) reported never crushing the capsule. Among those who ever crushed the capsule, logistic regression indicated that after controlling for demographic and smoking-related variables, females were more likely than males (Adj OR = 1.973, *p* = .015) to crush the capsule always or most of the time (vs. rarely or sometimes; Table 4). Likelihood of more frequent crushing of the capsule decreased with age (*p* = .001), with those aged 55+ less likely than those aged 18–54 years to crush the capsule always or most of the time (Adj OR = 0.204, *p* < .001). More frequent crushing was less likely among White non-British than White British (Adj OR = 0.423, *p* = .043) and among smokers with a higher score on the HSI (4–6) than those with a lower score (0–3; Adj OR = 0.310, *p* = .010).

**Table 4.** Logistic Regression of Association Between Demographic and Smoking-Related Variables and Frequency of Crushing the Capsule

Base: factory-made cigarette smokers (18 and over), who smoke capsule cigarettes, have smoked in the last month, and have ever crushed the capsule (344^a^, unweighted)	1 = Crush capsule always/most of the time (258)			
	0 = Crush capsule rarely/sometimes (86)			
	Adj OR^b^	95% CI lower	95% CI upper	*p*
Gender				
Male	ref			
Female	1.973	1.143	3.408	.015
Age group				.001
18–24	ref			
25–34 vs. 18–24	0.754	0.322	1.770	.517
35–44 vs. 18–34	0.609	0.299	1.238	.171
45–54 vs. 18–44	1.021	0.453	2.299	.961
55+ vs. 18–54	0.204	0.095	0.440	<.001
Social grade				
ABC1	ref			
C2DE	0.816	0.450	1.481	.504
Ethnicity				.042
White British	ref			
Other White background	0.423	0.184	0.974	.043
Other	0.421	0.163	1.088	.074
Heaviness of Smoking Index (HSI, 0 = light, 6 = heavy)				.006
0	ref			
1 vs. 0	0.572	0.242	1.354	.204
2 vs. (0–1)	0.543	0.261	1.131	.103
3 vs. (0–2)	0.615	0.308	1.225	.167
4–6 vs. (0–3)	0.310	0.127	0.759	.010
Whether planning to quit in next 6 mo				
No	ref			
Yes	1.105	0.639	1.960	.722
	Test of model coefficients: *χ*^2^ = 55.739, *df* = 13, *p* < .001. Nagelkerke *R*^2^ = 0.222.			
	Hosmer & Lemeshow *χ*^2^ = 5.283, *df* = 8, *p* = .727.			

95% CI = 95% confidence interval; Adj OR = adjusted odds ratio; ref = reference category.

^a^15 missing cases because of missing data across social grade, ethnicity, and Heaviness of Smoking Index where cell sizes were too small to include the “not stated responses” as separate categories.

^b^adjusted for all other variables in the model.

### Reasons for Using Capsule Cigarettes

Among those who smoked a capsule brand, the most common reasons for doing so were better taste (52%), a perception of them being smoother on the airways (41%), a desire for a choice of flavors (32%), enjoyment of clicking the capsule (25%), and because they are more interesting than regular cigarettes (21%). Almost one in seven capsule smokers indicated that their brand only has capsule variants (15%). The least common reason was novelty (9%), see Table 5.

**Table 5.** Reasons for Using Capsule Cigarettes

Base: factory-made cigarette smokers (18 and over), who smoke capsule cigarettes and have smoked in the last month (*N* = 454, weighted)	Total (*N* = 454)	
	*n*	%
They taste better than regular cigarettes	236	52
They are smoother on my airways (ie, throat and lungs) than regular cigarettes	185	41
I like having a choice of flavors	146	32
I enjoy clicking the capsule	114	25
They are more interesting than regular cigarettes	97	21
My brand only has capsule cigarettes	70	15
My friends, family, or work colleagues use or recommended them	50	11
They are novel	40	9
Other	28	6
Don’t know	20	4

### Perceived Harmfulness of Their Brand

Most (62%) of the sample did not perceive their brand to differ in harm relative to other brands. One-fifth (21%) did not know how their brand compared with other brands in terms of harm, while 12% thought their brand was a little less harmful than other brands, and 5% thought that their brand was a little more harmful. Perceptions of harm did not differ significantly among capsule and noncapsule smokers.

## Discussion

We found that approximately one in eight past month factory-made cigarette smokers in our sample preferred a brand with a flavor capsule in the filter. Tobacco companies highlight innovation as instrumental to their continued success^19^ and point to flavor capsules as an innovation driving growth around the world.^10,20^ Tobacco company internal documents highlight the importance of innovation for younger people,^21^ and consistent with this, we found that the use of capsule cigarettes decreased with age, being greatest among younger adults, as has been found in other high-income countries.^16,17^

We explored how these products are being used, with most capsule smokers indicating that they always (51%) crushed the capsule. Past research found that 52% of smokers always crushed the capsule in Mexico, 37% in the United States, and 30% in Australia.^16^ We also found that 9% never crushed the capsule, similar to the United States (5%), Australia (16%), and Mexico (4%).^16^ As with previous research,^16^ females were more likely to crush capsules more frequently, as were younger and middle-aged smokers, White British, and those with a lower score on the HSI. While we never specifically explored the factors underlying crushing the capsule, and future research doing so would be of value, in terms of women being more likely than men to crush the capsule more frequently, qualitative research in Scotland found that young women perceived capsule cigarettes to be fresher on their breath and have a less aversive and better disguised smell^11^; sensory characteristics such as aroma have been linked to appeal for women.^22^

We found that taste, choice of flavors, and enjoyment of clicking the capsule were the most prominent reasons for smoking capsule cigarettes. These results are generally consistent with tobacco industry reports which suggest that the main appeal of these products is a combination of taste, control, and personalization.^23–25^ That taste was the most prevalent reason for use in this study was expected, given the importance of taste in product choice, and is consistent with past research which suggests that capsule cigarettes are considered better tasting than regular cigarettes.^12,16^In terms of liking having a choice of flavors, it is argued that the flavor-on-demand allowed by capsule cigarettes helps them to compete with e-cigarettes,^9,23^ another novel product that has gained acceptance in the UK market. With respect to the enjoyment of clicking the capsule, this type of interactivity has been previously found to appeal to, or be targeted toward, young adults.^12,13^ That preference for capsule cigarettes decreased with age may help to explain why interactivity is a prominent reason for use; for younger people, interactivity is a prominent feature of their digital world, with Savelli et al.^26^ highlighting the link between the packaging of capsule cigarettes and digital technology and interactivity.

We did not find any evidence that capsule use was related to perceptions of reduced harm,^23^ consistent with qualitative research in Scotland^12^ and a survey in the United States,^17^ but not with a different survey in Mexico and the United States.^16^ As such, it is not clear why capsule use was more prevalent among those planning to quit smoking in the next 6 months compared with those not planning to quit, although as with mentholated tobacco in cigarettes, smoothness of taste may be a factor. A perception that capsules were smoother on the airways was the second most common reason for using these products, and it may be that this smoothness provides a subconscious sensation of reduced harm, which may appeal to those who intend to quit but are looking for reassurances about the harms from smoking.^27^ It may however be that motivations for using flavored capsule cigarettes differ from those for flavored noncapsule cigarettes, with conscious perceptions of reduced harm not being a key driver for capsule use. Our study provides some evidence for this, given that having a choice of flavors and the enjoyment of clicking the capsule were key reasons for use.

Despite the global success of flavored capsule cigarettes, few countries regulate their sale or ban flavored tobacco products. Articles 9 and 10 of the Framework Convention on Tobacco Control (FCTC) recommend a ban on flavors as they help increase the attractiveness and palatability of tobacco products^28^; our findings provide some support for this. To date, however, only Ethiopia, Uganda, Senegal, Canada and Brazil have banned all flavored tobacco products, although at least 30 European countries (European Union member states, Turkey and Moldova)^29^ will have done so by May 2020 as a result of the Tobacco Products Directive (TPD).^30^ For countries considering implementing a flavor ban and wanting to ensure that this encompasses flavors within capsules, which are not specifically mentioned in Articles 9 and 10 of the FCTC, they may wish to consider the wording of Article 7(7) of the TPD. Article 7(7) of the TPD explicitly prohibits “tobacco products containing flavourings in any of their components such as filters, papers, packages, capsules or any technical features allowing modification of the smell or taste of the tobacco products concerned or their smoke intensity.”^30^

In terms of limitations, while our sample was weighted to represent the national profile of smokers aged 16 and over in the United Kingdom, the YouGov panel may not be representative of the general population of smokers. For online panels, disparities in internet access are likely to lead to an under-representation of smokers from lower socioeconomic groups, among whom smoking is disproportionately concentrated, and also older aged participants, although this group typically have the lowest smoking rates.^31^ Nevertheless, internet penetration is high in the United Kingdom, with 89% of households having internet access in 2016 and 82% of adults reporting daily or almost daily internet use.^32^ It has been argued that online research also has the advantage of offering a more accurate reflection of individuals’ beliefs, given evidence of lower social desirability biases when not responding to another person.^33^ In addition, our estimated prevalence of preference for flavor capsule brands is generally consistent with industry reports,^10^ and our study provides a relatively nuanced portrait of this growing segment of the market that appears to be attracting younger people in particular. Future studies are needed to assess whether the appeal of this product is particularly strong among adolescents, as found in other countries.^14,15^

To defend market shares and capture new markets, innovation and providing consumers with more choice are considered increasingly important for tobacco companies.^34^ The World Health Organization describes these efforts to influence and expand markets as a major threat to public health,^35^ and in terms of capsule technology, further innovation seems inevitable. For instance, while we focused exclusively on factory-made cigarette smokers, given that there were no capsule brands of rolling tobacco on the UK market, capsule filters for rolling tobacco will almost certainly be introduced to the United Kingdom or other markets in the near future, with make your own cigarette tubes with filter capsules already available in some markets. Beyond capsules in the filter, a recent patent assigned to Philip Morris, United States is for a cigarette with a strip of microcapsules on the filter, where these can be peeled or scratched to release an odorant.^36^ Given the remarkable success of capsule cigarettes in the last decade, which seem to appeal to younger people in particular, then greater research is needed on these products and governments may wish to consider how these products are regulated.

## Funding

This work was supported by Cancer Research UK and British Heart Foundation (C312/A15192).

## Declaration of Interests

None declared.
